# Improving Accuracy and Efficiency of NEWS Chart Monitoring in a Forensic Mental Health Unit

**DOI:** 10.1192/bjo.2026.11451

**Published:** 2026-06-30

**Authors:** Chinwe Vivian Okeke, Kole Swapan, Ishwari Sharma

**Affiliations:** TEWV, Middlesbrough, United Kingdom

## Abstract

**Aims:**

To improve the accuracy and efficiency of NEWS monitoring by assessing flexibility for parameter recalibration and standardising documentation practices by March 2026.

To ensure NEWS parameters are safely and accurately adjusted for patients with atypical baselines (e.g. on psychotropics), in line with TEWV policy.

**Methods:**

PDSA Cycle 1 – Completed November 2025

Plan: To understand current NEWS monitoring practice and identify gaps related to accuracy, recalibration flexibility, and efficiency. This was following discussion with colleagues currently working with the secure inpatient services.

Specific objectives:

- Assess how frequently NEWS scores are documented electronically and on paper.
- Determine whether ward systems allow recalibration of NEWS parameters.
- Identify whether NEWS charts are being used for non-NEWS purposes.
- Compare practice against TEWV NEWS Policy and NICE guidance.

The expected outcome was to highlight inconsistencies and identify initial improvement opportunities.

Do:

- Collected data from five forensic wards using a structured yes/no/na audit tool.

Obtained data on:

1. Electronic NEWS recording.

2. Paper NEWS recording.

3. Ability to adjust/recalibrate parameters.

4. Misuse of charts.

• Converted findings to percentage compliance to allow cross-ward comparison.

Study: Key findings from Cycle 1:

- 72% of wards recorded NEWS electronically.
- 80% recorded NEWS on paper, showing duplication.
- 0% electronic recalibration available.
- 100% of paper charts allowed manual parameter adjustment.
- 28% of wards used paper charts for non-NEWS tasks.
- 0% misused electronic charts.

Interpretation:

- There is a reliance on paper charts for flexibility.
- The electronic system is inflexible, which may affect clinical accuracy.
- Documentation duplication results in inefficiency and possible errors.
- Paper misuse indicates lack of standardisation.

Act: Based on Cycle 1, the project team decided to:

- Present and disseminate findings to the ward teams and managers.
- Develop guidance on when and how to adjust NEWS parameters for atypical baselines.
- Explore options with digital teams for electronic NEWS customisation.
- Plan staff education on appropriate chart use.
- Prepare tests of change for Cycle 2.

These findings shaped the next phase of improvement work.

**Results:**

- 72% of wards recorded NEWS electronically.
- 80% recorded NEWS on paper, showing duplication.
- 0% electronic recalibration available.
- 100% of paper charts allowed manual parameter adjustment.
- 28% of wards used paper charts for non-NEWS tasks.
- 0% misused electronic charts.

Summary of Wastes: Duplication of documentation, inability to recalibrate electronically, misuse of paper charts, risk of inaccurate scoring, and reliance on inefficient workarounds all contribute to waste in time, motion, accuracy, and staff expertise.

**Conclusion:**

The data show good overall compliance with NEWS recording but significant limitations in electronic flexibility and non-standardisation of paper chart use.

To align with TEWV policy and NICE guidance, a structured recalibration approach and system improvements are required.

